# The Effect of Task-Oriented Training on Upper-Limb Function, Visual Perception, and Activities of Daily Living in Acute Stroke Patients: A Pilot Study

**DOI:** 10.3390/ijerph19063186

**Published:** 2022-03-08

**Authors:** Wonho Choi

**Affiliations:** Department of Physical Therapy, Gachon University, Incheon 21936, Korea; whchoi@gachon.ac.kr; Tel.: +82-32-820-4423; Fax: +82-32-820-4420

**Keywords:** activities of daily living, acute stroke, table-top activity, task-oriented training, upper-limb function, visual perception

## Abstract

This pilot study aimed to investigate the effects of task-oriented training on upper-limb functioning, visual perception, and activities of daily living (ADL) in acute stroke patients. Of 20 participants, 10 were randomly assigned in a 1:1 ratio to experimental and control groups. Task-oriented training and table-top activity training were implemented for 6 weeks. Change in upper-limb functioning was assessed with the Manual Function Test (MFT); visual perceptual skill and ADL performance were evaluated using the Motor-Free Visual Perception Test—Vertical (MVPT-V) and Korean Modified Barthel Index (K-MBI), respectively. There was a significant interaction in the MFT and MBI score between the group and time (*p* < 0.05), but the group effect was not significant (*p* > 0.05). The MFT and MBI score significantly increased in both groups after the intervention (*p* < 0.001), but the effect size was greater in the task-oriented training group than the table-top activity training group. No significant interaction with MVPT-V score was found between the group and time (*p* > 0.05), and no statistical group difference was found either (*p* > 0.05). Both groups showed significant improvement in their MVPT-V score after the intervention (*p* < 0.001). The pilot study findings demonstrate that both task-oriented training and table-top activity training are effective in recovering upper-limb function, visual perception, and ADL in acute stroke patients.

## 1. Introduction

In 2018, stroke was the fourth leading cause of death in Korea [1], and the annual incidence shows an increasing trend [2]. Stroke refers to a neurological disorder caused by cerebrovascular injury [3]. It is defined as a sudden brain attack that causes partial or complete brain dysfunction due to the brain’s blocked or ruptured blood vessels [4]. The primary symptom of a stroke is hemiparesis. Additionally, stroke adversely affects sensations, motor function, perception, cognition, and language, depending on the location, etiology, and infarct volume. A study reported that 85% of stroke patients had hemiparesis, and more than 69% showed upper-limb dysfunction [5].

Visual perception is a process in which the central nervous system integrates visual information and helps withdecision-makingg by converting information arriving at the retina to cognitive concepts so that individuals can adapt to the environment [6]. It aids in making accurate judgments regarding an object’s size and form and spatial relationship. Vision means the ability to see with accuracy and interpret visual stimuli [7,8]. Most activities in daily life are closely linked to visual perception. Visual perception is used in almost all activities, e.g., walking, eating, spatially orienting oneself, and writing, and it is also a prerequisite for perception and cognition and influences postural control and motor planning [9]. The anticipation of information essential for adequately adapting to an environment is also made possible through vision [10,11]. Visual perceptual disorder is defined as a decreased ability to process unconscious inference from the information of visual images generated by interactions between the frontal and parietal lobes [12]. A considerable percentage (35–75%) of patients with brain injury were inferred to experience impaired visual perception, which limits the ability to perform activities of daily living (ADL) independently [12,13]. Many visual perceptual disorders were shown to be associated with stroke-related visual impairments, which can also involve loss of visual field, ocular motility disorders, and strabismus [13,14].

In general, upper-limb dysfunction not only causes problems in performing tasks requiring fine motor skills (such as everyday tasks including personal hygiene activities, eating, and handwriting) but also makes it very difficult to perform tasks requiring gross motor skills (such as balancing, walking, and self-protective reflexes) [15]. Upper-limb dysfunction lowers stroke patients’ quality of life (QoL). It restricts their ability to live independently in society because it impairs most fundamental ADLs that are essential for individuals to live independently (such as crawling, balancing, walking, writing, eating, washing, and manipulating objects) [9]. Hands and arms are used in an extremely wide range of tasks performed at the workplace and used to communicate. Furthermore, they play a crucial role in various cognitive activities and motor functions [11]. In stroke patients, the recovery of upper-limb functions essential for independently performing ADLs may be regarded as critical as the recovery of walking [16]. Typically, the highest recovery rate is noted within three months after a stroke, and the progress between 3 and 6 months after the stroke is gradual [17,18]. This suggests that timely treatment is critical in optimizing functions in stroke patients and stresses the importance of early treatment [19].

In rehabilitation, a goal of therapeutic training is to help patients try to use the affected side and voluntarily perform motor functions, and there are a variety of physical intervention approaches [20]. Of those, task-oriented training is reported to be effective in improving the functional motor skills required for performing ADLs, according to several recent studies conducted in stroke patients [21,22,23]. Task-oriented training can help patients increase their ability to perform ADLs. The training consists of diverse functional activities, presenting a more efficient treatment approach [24]. Task-oriented training is a therapeutic model based on a system theory of motor control which regulates the movements of organisms with a nervous system, including reflexes [15,25]. It uses a functional approach in the rehabilitation of neurological patients and teaches the patients task-specific strategies to help them adapt to changing environments [26,27]. The approach involves having patients practice a skill essential for achieving the goal of a task to facilitate problem solving by enhancing their ability to adapt to various situations and developing an effective reward strategy [26,28]. In addition, for maximal learning, the approach involves behaviorally motivating patients by using tasks related to their personal daily life and emphasizes the interaction between patients and their environment [24]. A patient-centered approach, rather than a therapist-centered approach, is used in task-oriented training, and meaningful tasks are selected as an essential component [29]. A therapeutic goal of task-oriented training is the maximum recovery of motor functioning through the proactive performance of activities using the upper limb on the affected side rather than on the intact side [30]. Van Peppen et al. stated that repetitive and focused task-oriented training improved the recovery of upper-limb functioning and could enhance the motor pattern, dexterity, and agility in the upper limb [31]. Lee also reported that task-oriented circuit training improved upper-limb functions, ADL performance, and self-efficacy in expressing confidence [32].

As discussed above, much research has been performed to investigate the effect of task-oriented training in stroke patients [29,30,31,32]. However, task-oriented training in the previous studies focused on achieving a functional task in the therapy room environment rather than performing the activities patients could undertake in daily life, thus making it difficult to generalize the findings. Accordingly, this pilot study aimed to investigate the effects of task-oriented training on the improvement of upper-limb functions, visual perception, and ADL performance in acute stroke patients by designing the training based on the types of tasks that anyone needs to perform in daily life. We hypothesized that task-oriented training would have a more significant effect on upper-limb functions, visual perception, and ADLs compared to table-top activity training, which is most commonly used in general occupational therapy in acute stroke patients.

## 2. Materials and Methods

### 2.1. Ethical Approval

The study was conducted according to the guidelines of the Declaration of Helsinki and approved by the Gachon University Institutional Review Board (1044396-202102-HR-034-01). All participants signed a statement regarding informed consent before beginning the study.

### 2.2. Subjects

A total of 27 acute stroke patients voluntarily participated in the study, and 3 patients who did not meet the inclusion criteria were excluded. The inclusion criteria of the study participants were: patients diagnosed with a stroke within three months based on CT and MRI findings. The following types of patients were excluded: patients with cognitive impairment or dementia with a Korean version of Mini–Mental State Examination (K-MMSE) score of 20 or lower who were unable to understand instructions; patients with a score of 4 or higher on the National Institutes of Health Stroke Scale (NIHSS); patients with orthopedic disorders; patients who could not be evaluated or treated because of the inability to understand oral instruction due to language disorders or other reasons; and patients with psychiatric disorders.

### 2.3. Procedure

A total of 24 patients were randomly assigned into either a task-oriented training group (*n* = 12) or a table-top activity training group (*n* = 12) using simple randomization methods with a computer-generated randomized table of numbers created before data collection.

**Figure 1:**
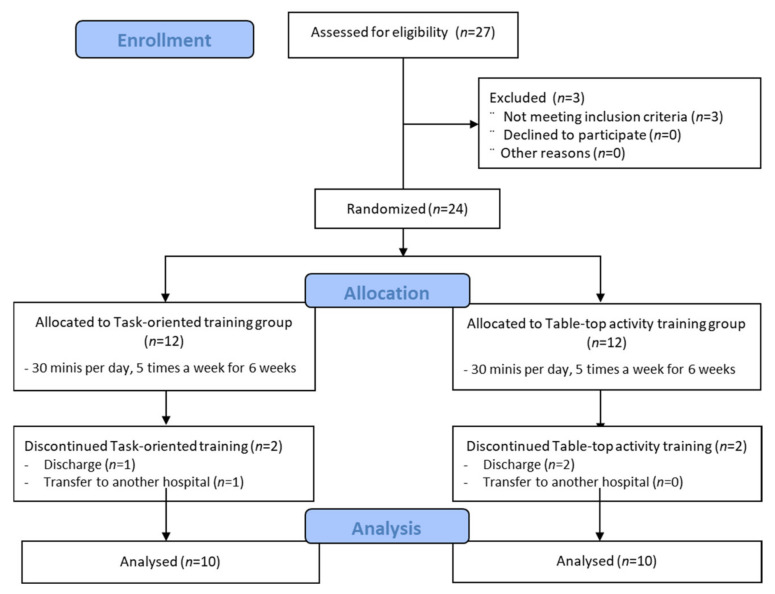
Flow diagram.

When the subjects arrived, general anthropometric characteristics, such as height, weight, and body mass index, were measured before the beginning of the experiment. Baseline measurement for Manual Function Test (MFT), Motor-Free Visual Perception Test—Vertical (MVPT-V), and Korean version of Modified Barthel Index (K-MBI) were measured. Afterward, both groups underwent intervention for 30 m a day, five times a week, for 6 weeks. The interventions were provided by two occupational therapists with more than 7 years of experience. Measurements of MFT, MVPT-V, and K-MBI were conducted after 6 weeks of intervention in both groups (Figure 1).

### 2.4. Outcome Measurements

#### 2.4.1. Manual Function Test

The MFT is a test used to assess a change in upper-limb functioning due to stroke. It can measure a change over a short duration during neurological recovery and is widely used in evaluating upper-limb functions and motor skills. The upper-limb function test in stroke consists of eight items, of which four pertain to upper-limb exercises, two to handgrip, and two to finger manipulation. The maximum total score is 32 points. The test is performed on both sides to predict the level of functional recovery of the upper limb on the affected side. In this study, the Manual Function Score was used [33].

#### 2.4.2. Motor-Free Visual Perception Test—Vertical

The MVPT-V is a standardized test used to evaluate visual perceptual skills in children and adults. It does not involve motor skills and thus can also be used for assessing patients with impaired motor function. Additionally, the test takes a shorter time to administer than other trials [34]. The MVPT-V consists of 36 items across five categories: spatial relationship, visual discrimination, figure–ground perception, visual closure, and visual memory. A point is assigned for each item if the patient answers correctly [35]. Additionally, this test can evaluate visual perceptual skills without using motor function and perceptual skill in stroke patients with hemi-spatial neglect [36].

#### 2.4.3. Korean-Modified Barthel Index

The K-MBI, which translated from the MBI developed by Shat et al., (1989) [37] by Jeong et al., (2007) [38], was used to evaluate the activities of daily living (ADL) of stroke patients. This consists of 10 items describing ADLs and mobility that were scored to measure the degree of assistance required by an individual. Each item is rated on a 5-Likert scale, with weights added according to the item, and the total score is 100. The higher the total score, the more independently the patient can perform ADLs. The earlier study established that reliability between evaluators of K-MBI was 0.93, and the reliability within the evaluator was 0.87 [38].

### 2.5. Intervention

#### 2.5.1. Task-Oriented Training Program

Table 1 illustrates the task-oriented movement therapy program used in the current study. To improve functional performance in patients by using tasks performed in daily life, those with a difficulty level between 0.2 and 0.7 that could be performed by any patients in the hospital and at home were selected from among the standardized tasks listed in the Assessment of Motor and Process Skills. The tasks were chosen based on patient counseling and confirmed by a professor with expertise in occupational therapy and two occupational therapists in their seventh year of practice. To control the difficulty level according to patient ability, patients were instructed to use both hands or only the affected hand. If a task was easy, the task in the next difficulty level was given to increase the time for task performance. Additionally, task difficulty was controlled by varying the size and weight of the object used in the task or changing the environment. Patients performed each item for 5 m, with a 1 m preparation time between tasks.

#### 2.5.2. Table-Top Activity Training Program

Table 2 illustrates the table-top activity training used in the current study. To improve upper-limb functions, therapeutic tools frequently used in occupational therapy were selected according to the definition of table-top activities. The task difficulty was based on patient performance level. Therapists who performed the intervention and treatment duration and frequency were identical to those in the task-oriented training program.

### 2.6. Statistical Analysis

IBM SPSS 26.0 software (IBM, Armonk, NY, USA) was used to analyze the data. Data were summarized as means and standard deviations (SDs) for continuous variables and frequencies and percentages for categorical variables. The Kolmogorov—Smirnov test was used to examine the assumption of normality of the continuous variables. Chi-squared test and independent t-test were performed to compare the general characteristics of participants at baseline. Repeated measures analysis of variance (RM ANOVA) was conducted to determine whether there was the group-by-time interaction effect of interest. When a significant interaction was found, an independent t-test was performed to compare the differences at pre- and postintervention between the two groups. A paired t-test was also conducted to compare outcome variables before and after the intervention in each group. Then, an effect size of Cohen’s d was used to identify how large the mean difference between before and after the intervention in each group. The level of statistical significance was set to α = 0.05.

## 3. Results

Of a total of 24 study participants, 4 (2 each in the experimental and control groups) dropped out due to hospital discharge or transfer to another hospital. Thus, the data for the remaining 20 participants were included in statistical analyses. The general characteristics of the participants are described in Table 3.

Table 4 shows the outcome variables before and after the interventions between the two groups. There was a significant interaction in mean MFT score between the group and time (*p* < 0.001), but the main effect of the group was not significant (*p* = 0.251). There was no significant difference between the groups at baseline (*p* = 0.741), while a significant difference was found after the intervention between the groups (*p* = 0.019). The MFT score significantly increased from 56.6 ± 12.9 to 79.4 ± 11.6 (*p* < 0.001) in the task-oriented training group, and the MFT score in the table-top activity training program also significantly increased, from 58.4 ± 11.0 to 65.0 ± 13.4 (*p* < 0.001).

Similarly, a significant group-by-time interaction effect was found in the mean MBI score (*p* = 0.013). The main effect of the group was not significant (*p* = 0.657), and no significant differences between the groups were found before or after the intervention (*p* = 0.615 and 0.150, respectively). The MBI score in the task-oriented training group significantly improved, from 55.9 ± 14.4 to 80.9 ± 12.3 (*p* < 0.001). Likewise, the score in the control group significantly increased, from 59.2 ± 14.5 to 72.5 ± 12.6 (*p* < 0.001).

Regarding the total pre- and post-intervention MVPT-V scores, no significant interaction was found between the group and time (*p* = 0.141), and no statistical group difference was found either (*p* = 0.735). Both groups showed significant improvement in the MVPT-V score after the intervention (27.8 ± 2.0 to 32.7 ± 2.5, *p* < 0.001 and 28.3 ± 1.3 to 31.7 ± 1.9, *p* < 0.001, respectively).

## 4. Discussion

Task-oriented training is a form of therapy for stroke patients. It is based on motor learning theory and aims to help patients adapt to the environment [29]. Unlike the treatment approach involving repeated training for single movements, in task-oriented training, functional tasks are used, and patients are trained to perform the tasks effectively and efficiently through the interaction of connected body parts for problem solving [39]. Task-oriented training has been demonstrated to be an efficient therapeutic approach for stroke patients because it consists of tasks that help improve ADL performance and effectively provides patients with training for diverse functional skills [40]. A previous study reported that task-oriented training based on ADLs improved upper-limb functions, ADL performance, and QoL in stroke patients [41]. Sung also showed that selective task-oriented training based on the occupational therapy practice framework effectively increased stroke patients’ daily activity performance and QoL, demonstrating the clinical effect of the training [42].

Both active and passive interventions that preserve active exercise, task-oriented training, and muscle extensibility have been emphasized to improve the motor function of stroke patients. It has been reported that this training maximizes functional performance and temporarily induces brain restructuring [43]. Additionally, upper-limb movements require the control of both the proximal and distal segments. After a stroke, however, recovery is faster in the proximal than in the distal segments; hence, dexterity is markedly reduced, making functional use of the upper limb in daily life laborious [44]. Upper-limb dysfunction after a stroke limits ADL performance and decreases patients’ QoL and social participation. Therefore, it is necessary to perform interventions that might aid the recovery of upper-limb functioning in stroke patients [45].

Previous research reported that the task-oriented training group showed a significantly greater improvement in an upper-limb function test than the biofeedback system training group [46]. However, another study reported that task-oriented training and control groups (which received repetitive physical therapy for the upper limb) showed significant improvement in an upper-limb function test [47]. Similarly, our results show that both groups showed significant improvement in upper-limb function. There was no significant group difference, but the magnitude of the improvement was larger in the task-oriented training group (*d* = 1.859) than table-top activity training (*d* = 0.538) after 6 weeks of intervention. Brain activation pattern as assessed by functional MRI was presented sporadically regardless of the lesion site in the stroke patients. However, it was specifically identified in the contralateral primary motor area and perilesional area after task-oriented training for upper limbs [48]. This restructured cortical region plays a vital role in the motor regulation of the upper limb and is associated with an efficient increase in corticospinal tract function. It could explain the effect of improving motor function recovery and reorganizing the motor network.

Visual perceptual therapy influences the recovery of visual perception, ADL performance, and cognitive function in stroke patients [49]. Furthermore, they reported that it is difficult to exclude the impact of visual perception in daily life due to the complexity of the human visual system. Visual perceptual dysfunction causes difficulty in reading, writing, eating, driving, locating objects in space, and performing various activities required in the workplace [50]. Different interventional approaches may improve visual perception depending on patients’ symptoms. Currently, the representative interventional approaches include visual perceptual intervention in conventional occupational therapy and computer-based visual perceptual training. However, reviews of visual perceptual intervention approaches have rarely been conducted [51].

Furthermore, there is little evidence for the effects of intervention approaches in improving visual perception [51]. Regarding the criteria for treating visual perceptual dysfunction in stroke patients, the intervention approach may be determined based on patients’ performance levels and the extent of visual perceptual impairment. Two categories of intervention approach exist: an active approach to improve an impaired skill and a passive approach to provide an environment to enhance task performance [51]. Accordingly, both active and passive approaches were used in the current study, and the effect of task-oriented training in the recovery of visual perception in acute stroke patients was demonstrated. Regarding the MVPT-V, the test used to assess visual perceptual performance demonstrated that task-oriented training and table-top activity training showed significant improvement. Although the former showed a more remarkable improvement, by a mean of 1.5 points, the intergroup difference in MVPT-V scores was not significant. This finding is inconsistent with a previous study, which showed that task-oriented training was more effective than typical occupational therapy alone [52]. It is speculated that the task-oriented training in this study consisted of tasks focused on motor skills, such as a table-top activities, and diverse medical characteristics of stroke patients and the environment was not considered.

The K-MBI test used to evaluate ADL performance showed that the score significantly improved in both groups, but no significant group effect was found. In some ways, the study findings are not in line with previous study findings that ADL performance significantly improved in task-oriented training groups compared to the groups that received occupational therapy intervention or action observation therapy intervention [53,54,55]. In the previous studies, ADL was significantly improved only in the task-oriented training group, but table-top activity training was also influential in our research. The motor and perceptual functions affect ADL performance [49]. In this pilot, ADLs were also improved in both groups as both upper-limb function and visual perception increased in both groups. A positive effect on the recovery of acute stroke patients through both interventions in rehabilitation of acute stroke patients was expected.

However, the current study has limitations that need to be addressed. A follow-up measurement was not conducted to investigate the persistence of intervention effects. Additionally, even though there were no significant differences in types of strokes and affected sides between the groups, these variables could affect motor, visual, or cognitive function so these variables should be considered for further study. In terms of task selection, tasks were not prioritized for each patient by considering his/her impairments. Instead, the selected tasks were determined irrespective of patient needs. Thus, the study was not based on patient-centered tasks. Additionally, since this pilot study sample size was relatively small, future studies should investigate the long-term effects of a task-oriented training program with a sufficiently large sample by improving upon the limitations.

## 5. Conclusions

The pilot study findings demonstrate that both task-oriented training and table-top activity training improve upper-limb function, visual perception, and ADL in acute stroke patients. In particular, the task-oriented training showed a greater improvement of the upper-limb function, so it is recommended that task-oriented training be applied for upper-limb function in acute stroke patients. Further studies should involve administrating various task-oriented training programs in chronic stroke patients to investigate the effects of upper-limb functioning and visual perception on ADL performance.

## Figures and Tables

**Table 1:** Task-oriented movement therapy program.

Task	Task-Oriented Training Program
Taking out a drink from the refrigerator and pouring it into a cup	Open the refrigerator door, take out a liquid-containing bottle, put it on the counter next to the refrigerator, close the refrigerator door, and pour the drink into a cup. (The difficulty level was controlled by varying the size of the bottle and the volume of the drink.)
Washing a cup	Wash the cup using dish soap and a sponge. (The difficulty level was controlled by varying the cup or tumbler size.)
Cleaning with a vacuum cleaner	Clean the dust off a floor, curtain, and furniture. (The difficulty level was controlled by instructing patients to lift the vacuum cleaner while vacuuming the floor to vacuuming an item located at a higher level, such as a curtain.)
Folding towels and stacking them	Sit on a chair and fold towels on a table and stack them. (The difficulty level was controlled by instructing patients to fold towels using both hands or one hand and to sit or stand while performing the task.)
Using a pair of chopsticks	Use Edison training chopsticks to grab a block (1 × 1 cm), a bean, and a pin. (The difficulty level was controlled by progressively using Edison training chopsticks, wooden chopsticks, stainless steel chopsticks, and Chinese-style chopsticks with a thick end.)

**Table 2:** Table-top activity training program.

Task	Occupational Therapy Program
Stacking cones	Lift a cone with one hand and stack it on top of a fixed cone. (The difficulty level was controlled by using resistance bands of varying weights or instructing patients to sit or stand while performing the task.)
Stacking 3 × 3 cm blocks	Pick up blocks one at a time by using one hand and stack them. (The difficulty level was controlled by using resistance bands of varying weights.)
Tailwind	Use both hands to push the handles of an instrument up and down to the sounds of a metronome. (The difficulty level was controlled by varying the angle.)
Pinch exerciser	Take a pinch pin and put it on a rod. (The difficulty level was controlled by varying the resistance of the pinch pin (5 grades) and the diameter of the rod (3 grades).)
O’Connor Tweezer Dexterity Test	Pick up a pin (2.5 cm long) and put it in a 15 mm hole. (The difficulty level was controlled by using different tweezers.)

**Table 3:** General characteristics of the participants (*N* = 20).

	Task-Oriented Training (*n* = 10)	Table-Top Activity Training (*n* = 10)	*p*
Age (years)	54.4 ± 12.7	59.8 ± 8.29	0.277
Sex, Females, *n* (%)	2 (20.0)	3 (30.0)	0.606
Stroke type			
Infarction, *n* (%)	5 (50.0)	8 (80.0)	0.160
Hemorrhage, *n* (%)	5 (50.0)	2 (20.0)	
Affected side, left, *n* (%)	4 (40.0)	7 (70.0)	0.178
Onset (months)	1.2 ± 0.4	1.6 ± 0.5	0.074
K-MMSE (scores)	25.7 ± 3.5	27.0 ± 1.8	0.308
NIHSS	3.1 ± 1.1	2.5 ± 1.4	0.380
MFT	56.6 ± 12.9	58.4 ± 11.0	0.741
K-MBI	55.9 ± 14.4	59.2 ± 14.5	0.615
MVPT-V	27.8 ± 2.0	28.3 ± 1.3	0.646

Abbreviations: K-MMSE, Korean Mini–Mental State Examination; NIHSS, National Institutes of Health Stroke Scale; MFT, Manual Function Test; K-MBI, Korean modified Barthel index; MVPT-V, Motor-Free Visual Perception Test–Vertical.

**Table 4:** Outcome variables before and after the interventions between the groups (*N* = 20).

		Task-Oriented Training (*n* = 10)	Table-Top Activity Training (*n* = 10)	Group × Time Interaction F (p)	Main Effect of Group F (p)
MFT	Pretest	56.6 ± 12.9	58.4 ± 11.0	36.9 (<0.001)	1.40 (0.251)
	Post-test	79.4 ± 11.6	65.0 ± 13.4		
	d ^†^	1.859 *	0.538 *		
K-MBI	Pretest	55.9 ± 14.4	59.2 ± 14.5	7.65 (0.013)	0.20 (0.657)
	Post-test	80.9 ± 12.3	72.5 ± 12.6		
	d ^†^	1.867 *	0.979 *		
MVPT-V	Pretest	27.8 ± 2.0	28.3 ± 1.3	2.37 (0.141)	0.12 (0.735)
	Post-test	32.7 ± 2.5	31.7 ± 1.9		
	d ^†^	2.164 *	2.089 *		

Abbreviations: MFT, Manual Function Test; K-MBI, Korean modified Barthel index; MVPT-V, Motor-Free Visual Perception Test—Vertical. ^†^ Cohen’s d, the effect size for the comparison between two means, * significant difference between pre- and post-test at the 0.001 level.

## Data Availability

The datasets generated during the current study are available from the corresponding author on reasonable request.
